# Prediction of Electrical Resistance with Conductive Sewing Patterns by Combining Artificial Neural Networks and Multiple Linear Regressions

**DOI:** 10.3390/polym15204138

**Published:** 2023-10-18

**Authors:** JunHyeok Jang, JooYong Kim

**Affiliations:** 1 Department of Smart Wearable Engineering, Soongsil University, Seoul 06978, Republic of Korea; 2 Department of Materials Science and Engineering, Soongsil University, Seoul 06978, Republic of Korea

**Keywords:** conductive yarn, wearable technology, artificial neural network, multiple linear regression analysis, optimization design

## Abstract

This study aims to estimate the impact of sewing thread patterns on changes in the resistance of conductive yarns coated with silver paste. Firstly, the structure of the conductive yarns was examined, and various variations in the length and angle of individual sewing stitches were observed and analyzed through experiments. The results revealed that as the length of an individual stitch decreased, the width of the conductive yarn increased. Additionally, variations in the stitch angle resulted in different resistance values in the conductive yarn. These findings provide essential information for optimizing sewing patterns and designing components. Secondly, the comparison between models using multiple linear regression analysis and sewing neural networks was included to show optimized resistance prediction. The multiple linear regression analysis indicated that the stitch length and angle were significant variables affecting the resistance of the conductive thread. The artificial neural network model results can be valuable for optimizing sewing patterns and controlling resistance in various applications that utilize conductive thread. In addition, understanding the resistance variation in conductive thread according to sewing patterns and using optimized models to enhance component performance provides opportunities for innovation and progress. This research is necessary for the textile industry and materials engineering fields and holds high potential for practical applications in industrial settings.

## 1. Introduction

In recent years, conductive yarns have been widely used in smart wearable devices and smart clothing [1,2,3]. They have also been employed in wearable energy harvesting to transmit harvested energy [4,5] and used for signal transmission in various wearable sensors [6,7,8]. Conductive threads serve as a simple power delivery medium and have roles as sensors or actuators [9,10]. They have been extensively studied and applied in various fields, including using conductive coating fibers [11,12,13,14].

Conductive yarns used in different fields can be applied in various forms. Techniques such as weaving, adhesion, and sewing exist, with sewing being advantageous due to its simplicity and applicability to a wide range of fabrics [15]. However, an analysis of resistance variation is necessary when using sewing to utilize conductive yarns. The resistance of conductive threads can change depending on their form and external factors [16]. Resistance variation can result in power loss during power transmission [17]. When there is significant power loss due to the conductive yarn, it can lead to reduced energy efficiency and also impact signal transmission. Therefore, there is a need to study the resistance variation in conductive yarns in power transmission through conductive yarns in wearable devices. Specifically, there is a need to investigate the impact of embroidery patterns on conductive yarns when applied to wearable devices and how such effects influence resistance variation.

Furthermore, conductive yarns can be produced through different fabrication processes, such as sputtering for plating and coating with metallic solutions, resulting in structural variations. When coated with silver paste, conductive yarns exhibit a different surface structure compared to those covered through plating, with silver existing in particle form on the surface of the yarn. As a result, they do not exhibit the typical resistance variation pattern seen in conventional cables and instead can undergo resistance changes in various forms depending on the tension applied to the yarn [18,19,20,21,22]. This is essential for the design of wearable devices that demonstrate optimal power efficiency and enable their extension into sensor applications, as demonstrated in the paper. There have been numerous research efforts aimed at optimizing challenging predictions, particularly using MLR and ANN models, as seen in the study described. The objective has often been to select the model that offers the highest predictive accuracy. This has been particularly relevant in processes involving the incorporation of nanoparticles, which tend to introduce greater complexity to predictions. In recent times, research endeavors have been increasingly focused on using MLR and ANN models to predict and optimize variables that are challenging to predict, spanning not only material processes but also encompassing various aspects such as environmental factors and structural stability. This underscores the growing importance of these modeling approaches in tackling complex predictive challenges across diverse fields [23,24,25,26,27,28,29,30,31].

This study aims to analyze how the resistance of conductive yarns changes according to sewing patterns. The findings can easily be applied to wearable devices and smart clothing. Experiments will be conducted by varying the angles and stitch lengths, which are two variables that can occur in sewing, to observe how conductive yarns respond to these changes. The study will analyze and discuss the influence of conductive yarn variations on resistance. The final goal of this study is to compare the analysis using MATLAB ANN and multiple linear regression analysis in order to find a suitable optimized model. In the realm of smart wearables and smart clothing, conductive threads play a multifaceted role encompassing functions such as sensors, electrodes, and power transmission. Achieving the appropriate resistance values tailored to specific applications is of paramount importance. This is attributed to several critical factors, including the need for precise signal transmission, minimization of power loss during power transmission, enhancing the functionality of sensors (e.g., GF or gauge factor), and minimizing resistance in electrodes.

By leveraging the findings of this research, the optimization of resistance variations in conductive threads through simple sewing techniques holds the potential to be a game-changer. It could enable the seamless integration of conductive threads in various applications, promising more accurate signal transmission, reduced power losses, and enhanced functionalities across a wide spectrum of fields.

## 2. Materials and Experiments

### 2.1. Materials

**Figure 1:**
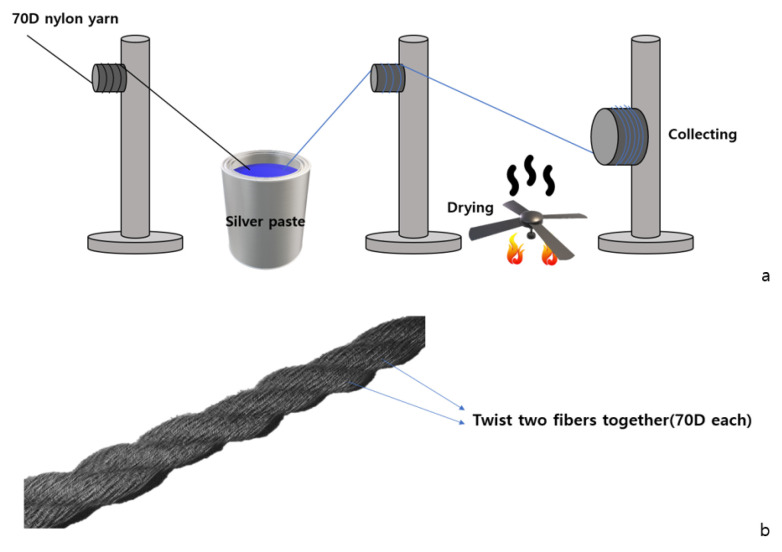
(**a**) Process of making conductive yarns. (**b**) A 2-ply conductive yarn.

Conductive yarn can be produced by coating yarn with a conductive solution and drying it [14]. As shown in Figure 1, the conductive yarn used in the experiment (Soitex, gyeonggi-do, Republic of Korea) was made by immersing 70D nylon fibers in a silver paste solution (silver particle content 72%, Yoo Sung Yeiin, Shanghai, China), drying them, and then twisting the two fibers together. Furthermore, the drying conditions involve 30 min hot air drying at 100 °C following 10 s immersion.

**Figure 2:**
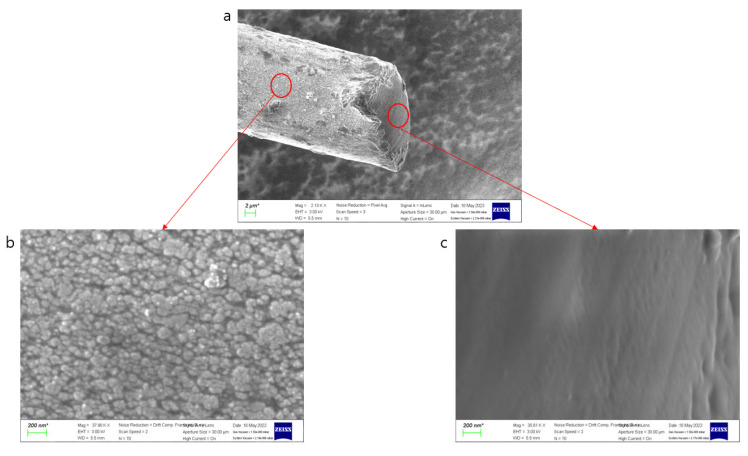
(**a**) SEM image of a single conductive fiber. (**b**) Silver particles coated on the surface. (**c**) Nylon comprising the core.

**Figure 3:**
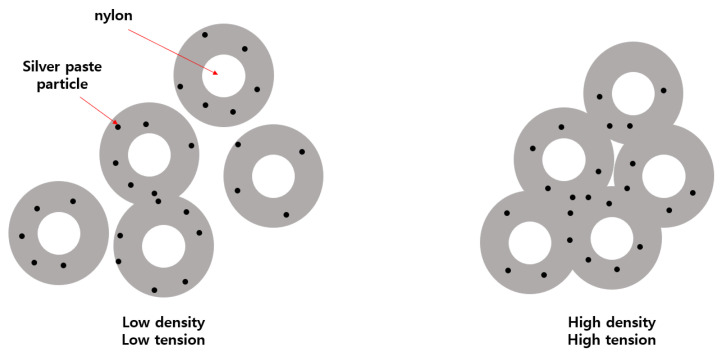
Changes in silver particles according to tension applied to fibers.

**Figure 4:**
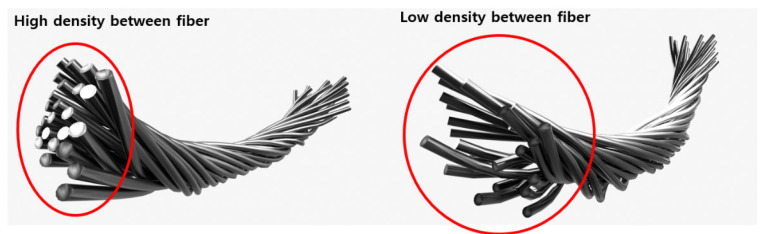
Difference in density between fibers.

**Figure 5:**
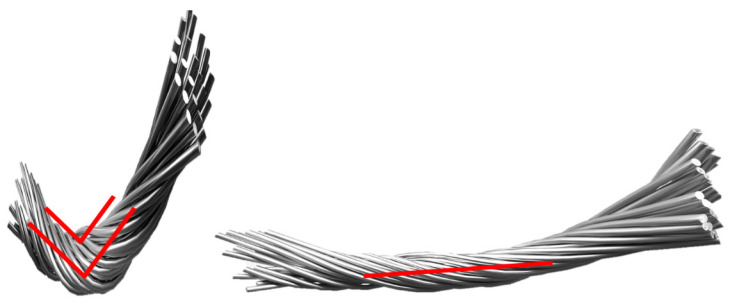
Comparison of electron conduction paths shortened by bending angle.

Using a scanning electron microscope (SEM), specifically the ZEISS Gemini SEM 300 from Oberkochen, Germany, we observed the surface of the conductive yarn coated with silver paste [32]. As can be seen in Figure 2, it exhibited a structure similar to the SEM image of silver paste coating. However, as indicated in Figure 2, the conductive yarn coated with silver by sputtering showed uniform crystalline silver coating, whereas the conductive yarn coated with silver paste exhibited empty spaces between silver particles on the surface. The variation in the resistance of silver-coated conductive yarn based on tension can be attributed to changes in the spacing between silver particles due to tension [16,33]. As shown in Figure 3, as tension increases, and the density between fibers becomes stronger, the spacing between silver particles, which exhibit conductivity, changes, resulting in variations in resistance [17]. The resistance decreases further when the spacing between conductive particles becomes even closer. Figure 4 illustrates the density differences among conductive yarn fibers, and Figure 5 demonstrates that resistance can vary depending on the angle formed during the sewing process. Electric current flows through the path with the lowest resistance. Furthermore, due to the voltage distribution law, it can manifest as a parallel combination of numerous resistances. Therefore, conductive yarn coated with silver paste can exhibit varying resistance depending on the sewing method, making it challenging to predict straightforwardly. However, when predictability is achieved, it offers the advantage of being suitable for sensor applications. Additionally, when used for power transmission, it has the potential to enable designs that optimize power efficiency.

### 2.2. Experiments

The length of a single stitch and the angle at which it deviates in sewing can influence the resistance of the straight conductive thread pattern on the surface by altering the spacing between coated particles. Therefore, the conductive yarn is embroidered by varying the stitch length and angle. The sewing was conducted using a Jack A4 sewing machine (Jack, Jiangxi, China), considering the machine’s specifications. The stitch length was varied from 1 mm to 5 mm in 1 mm increments. To account for the potential resistance variation due to the total stitch length, a uniform total length of 10 cm was chosen arbitrarily.

**Figure 6:**
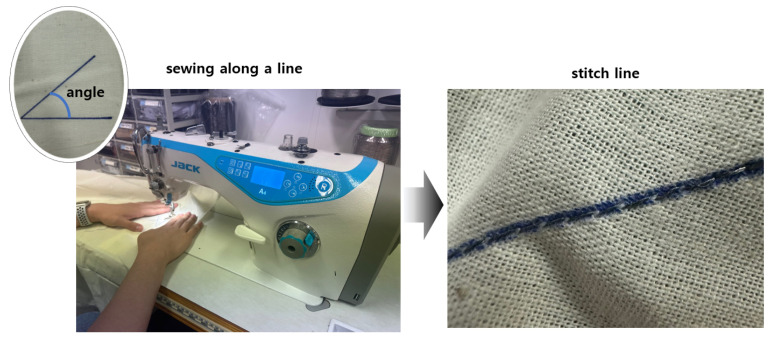
The entire process of sewing.

Additionally, the angle of the stitch was varied from 180° to 20° in 20° increments, starting from the center point. The upper thread used in the experiment was the conductive thread, and the lower thread was a regular 140D polyester thread (Hyosung, Seoul, Republic of Korea). The tension of the upper thread was fixed at the maximum value, ensuring it did not break. This was done because changes in the stress of the conductive yarn can affect its resistance, and by fixing the tension at the maximum, the phase shift between the upper and lower threads can be avoided when changing the stitch length. The general process for sewing is depicted in Figure 6. We conducted the experiments while maintaining uniform tension of the thread, consistent stitching through the fabric, and equivalent conductivity properties and total quantity of the bobbin thread.

The produced stitches were measured for width and length using an LCD electronic microscope (L910, Shenzhen, China). The length measurement of the width was used to investigate the fiber density variation based on the variables. Subsequently, the resistance was measured using an electronic engineering workstation, the Analog Discovery 2 (NI, Austin, TX, USA). To minimize measurement errors, the stitches were secured at the end using tweezers while measuring the resistance. Each stitch was produced in sets of 10, and the resistance was measured for 1 s at a rate of 100 Hz. A total of 1000 resistance data points were collected for each sample, and their average values were calculated.

Multiple regression analysis (MLR) is a statistical analysis technique used to model the relationship between a dependent variable and multiple independent variables. This method is used to understand and predict how the independent variables influence the dependent variable. Multiple regression analysis assumes a linear relationship among the independent variables and estimates the coefficients of the model using the least squares method. Such linear models are used to predict the values of the dependent variable based on the linear correlation between variables [34]. In MLR, the dependent variable is also known as the predictor, and the independent variables are referred to as predictors and can be expressed using the following equation:

(1) $$Y=\beta_{0}+\beta_{1}X_{1}+\beta_{2}X_{2}+\cdots +\beta_{n}X_{n}+\varepsilon$$

where Y represents the dependent variable, $X_{i}$ represents the independent variables, $\beta_{i}$ represents the estimated parameters, and *ε* represents the error term [35]. In this study, statistical analysis was performed using the MATLAB regression analysis tool.

Artificial neural networks (ANNs) are machine-learning algorithms inspired by the functioning principles of biological neural networks. ANNs consist of multiple neurons organized into input, hidden, and output layers. Each neuron receives input values, applies weights and activation functions, and calculates output values. ANNs are known for their ability to learn complex nonlinear relationships and are used for prediction and classification tasks in various problems. They are particularly effective in modeling complex interactions among multiple variables [36].

**Figure 7:**
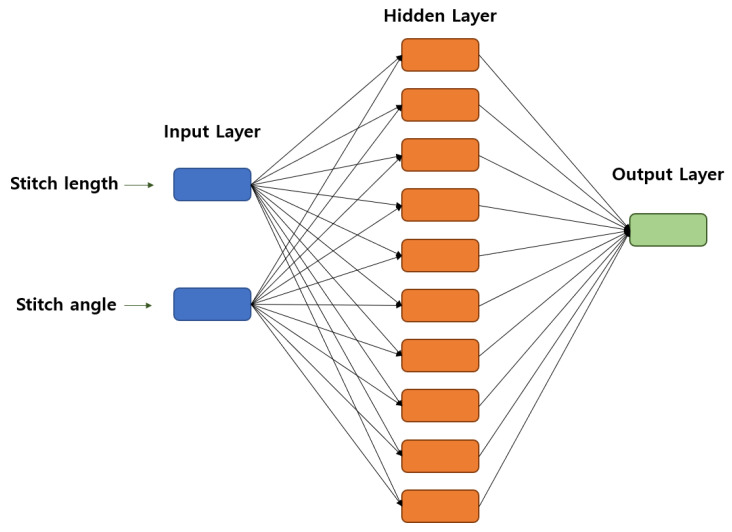
Architecture of the ANN model.

The neurons in the network are interconnected using weight factors ($W_{ij}$). A given neuron (*j*) in a layer receives information ($X_{i}$) from all neurons in the previous layer. The weighted sum of the connections and biases of the layer is calculated as ${net}_{j}$. Then, a mathematical function (f(.)) is applied to ${net}_{j}$ to calculate the output value ($y_{j}$), which is transmitted to all neurons in the next layer, and this process is formalized by Equations (2) and (3) [35]. The ANN used in this study is depicted in Figure 7.

(2) $${net}_{j}=\sum_{i=1}^{n}X_{i}W_{ij}-\theta_{j}$$

(3) $$y_{j}=f\left({net}_{j}\right)=\frac{1}{1+e^{-{net}_{j}}}$$

The number of neurons in the input layer corresponds to the number of input (independent) variables, and the number of neurons in the output layer is equal to the number of output (dependent) variables in a prediction problem based on cause-and-effect relationships. However, there is currently no clear rule for determining the number of hidden neurons, so the number of hidden layers and neurons is typically determined through a trial-and-error process. If the structure of an ANN model is too simple, the trained network may not sufficiently learn the relationship between the input and output. On the other hand, if the structure is too complex, the training of the network may overfit or fail to converge to the target error [37]. In this study, statistical analysis was conducted using the MATLAB Artificial Neural Network Fitting App (version 9.3.0). The hyperbolic tangent sigmoid transfer function (tansig) was used in the hidden layer, and the linear transfer function (purelin) was preferred as the activation function in the output layer. The Levenberg–Marquardt algorithm (trainlm) was used as the training algorithm, and the momentum backpropagation algorithm (traingdm) was used as the learning rule. The ANN training was stopped after 1000 epochs.

In this study, we aim to compare multiple regression analysis and artificial neural networks for predicting resistance optimization. The mean square error (*MSE*) and coefficient of determination (*R*^2^) were used for performance evaluation of the two models. *MSE* and *R*^2^ were calculated using the following equations:

(4) $$MSE=\frac{1}{N}\sum_{i=1}^{N}{\left(t_{i}-td_{i}\right)}^{2}$$

(5) $$R^{2}=1-\frac{\sum_{i=1}^{N}{\left(t_{i}-td_{i}\right)}^{2}}{\sum_{i=1}^{N}{\left(t_{i}-\bar{t}\right)}^{2}}$$

Here, $t_{i}$ represents the measured values of the experimental samples, $td_{i}$ represents the predicted values, $\bar{t}$ is the average of predicted values, and *N* represents the total number of samples.

MLR aims to model the linear relationship between resistance and independent variables, such as the length of a single thread and the angle of stitches. On the other hand, artificial neural networks have the ability to learn nonlinear relationships, making them more suitable for modeling resistance optimization with complex interactions. Through this comparison, we aim to derive the optimal resistance prediction model by determining which model exhibits superior predictive performance.

## 3. Results

**Figure 8:**
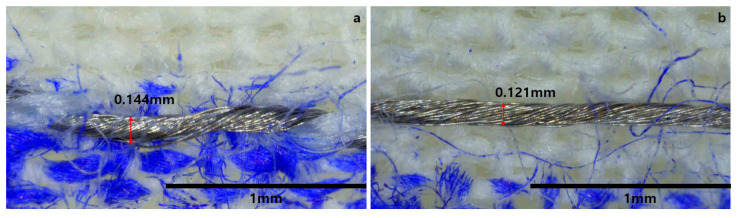
(**a**) Length of the sewing stitch is 1 mm. (**b**) Length of the sewing stitch is 5 mm.

At 180°, the width of a sewing stitch varies with the length of the stitch, as shown in Table 1. Additionally, as depicted in Figure 8, as the length of a sewing stitch decreases, it forms a convex shape, affecting the spacing between filaments and, consequently, the spacing between particles on the surface. Furthermore, we examined how much the spacing between silver particles can widen based on the length of the longest part of the width. This indicates that with the tension of the thread fixed, an increase in the length of a sewing stitch results in a higher fiber density between the threads. The reason behind this is that as the length of a sewing stitch decreases, the tension of the bottom thread becomes stronger, leading to a transformation in the appearance of both the bottom and top threads during sewing [38]. As the tension of the bottom and top threads becomes similar, the top thread exhibits less tightness compared to when the tension of the top thread is stronger [39,40].

**Figure 9:**
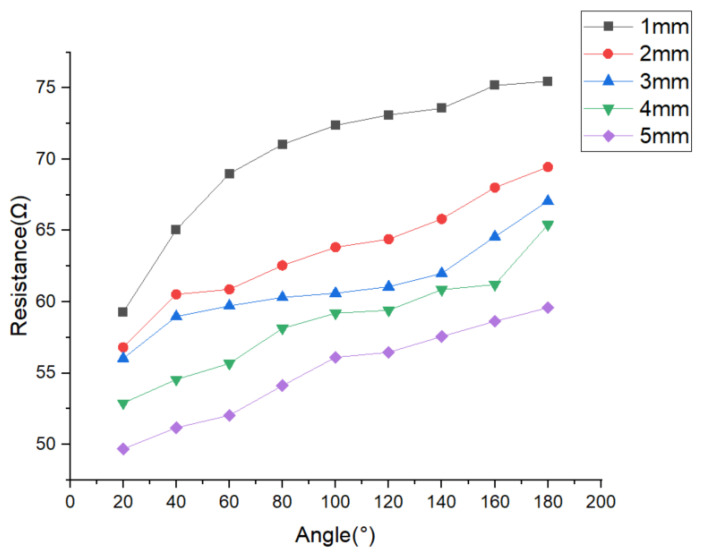
The resistance variation with the angle for each stitch length.

The resistance values for each sewing stitch sample are presented in Table 2. Furthermore, Figure 9 illustrates the resistance variation with the angle for each stitch length.

**Figure 10:**
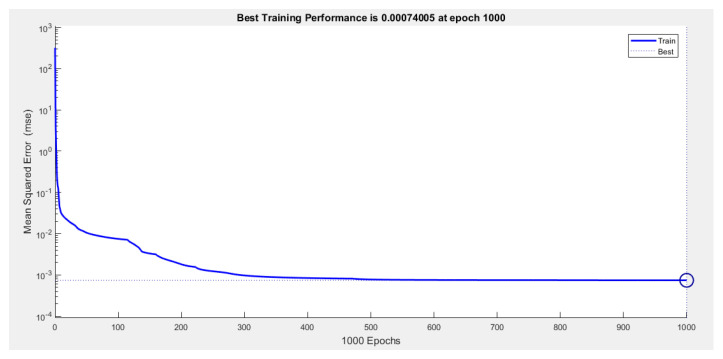
The variation in error with respect to the iterations of the ANN model.

In the analysis using the ANN, the MSE reached approximately 0.0074 after 1000 epochs. The variation of error with respect to the iterations of the ANN model can be observed in Figure 10.

**Figure 11:**
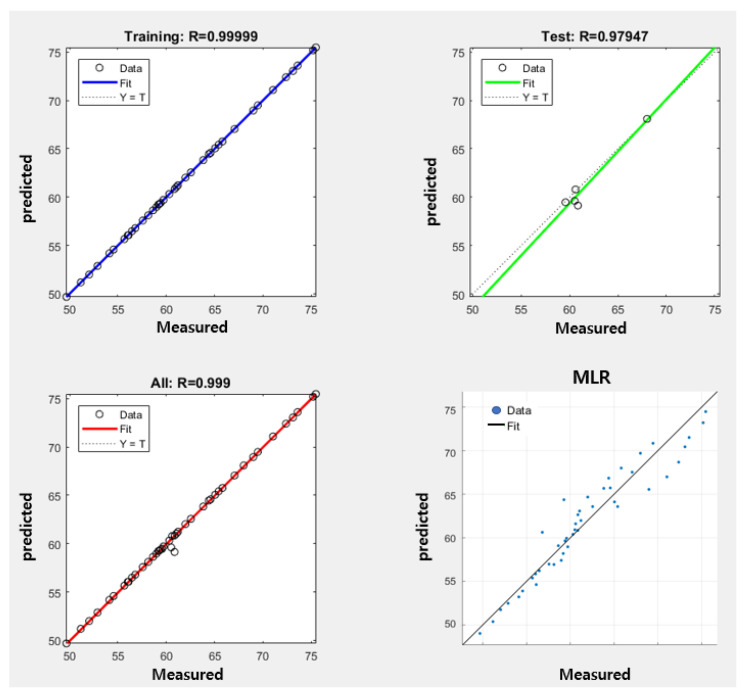
Variation in the predictions and measurements of the ANN and MLR models.

**Figure 12:**
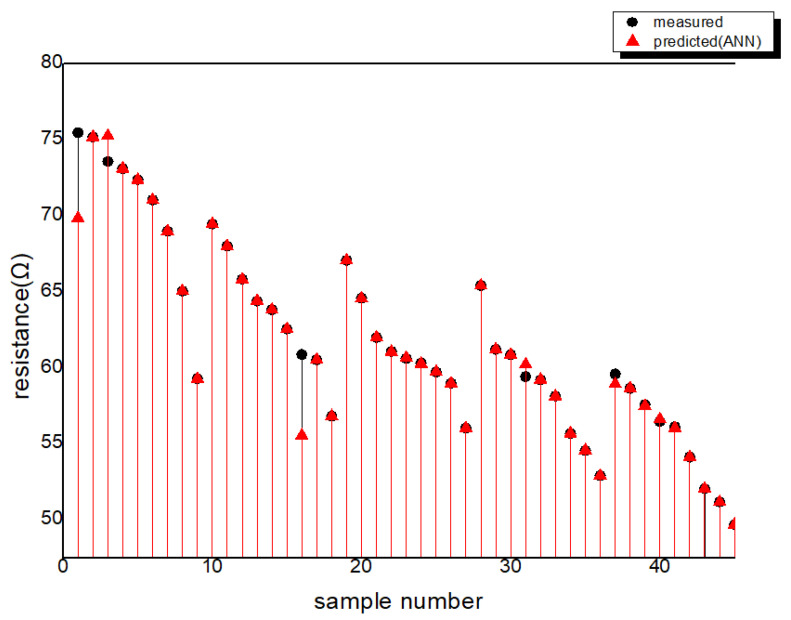
The distribution of the actual measured values and the values predicted by MLR.

**Figure 13:**
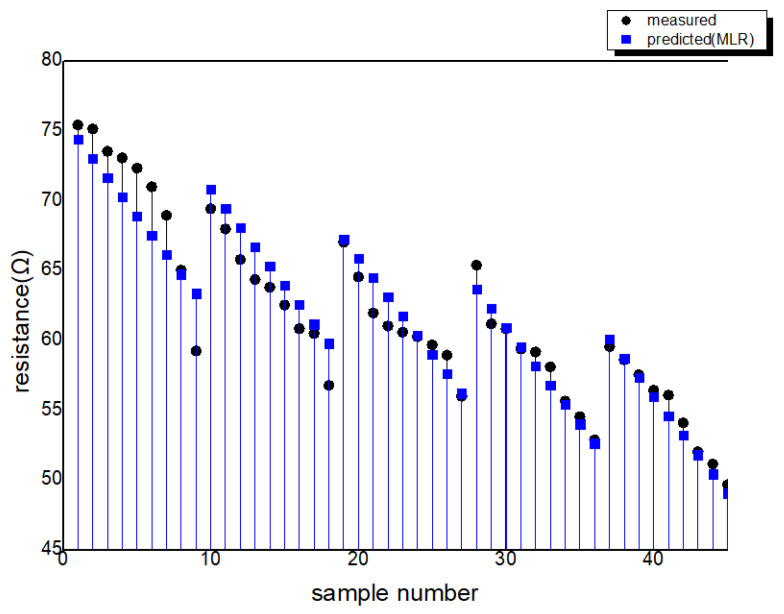
The distribution of the actual measured values and the values predicted by ANN.

**Figure 14:**
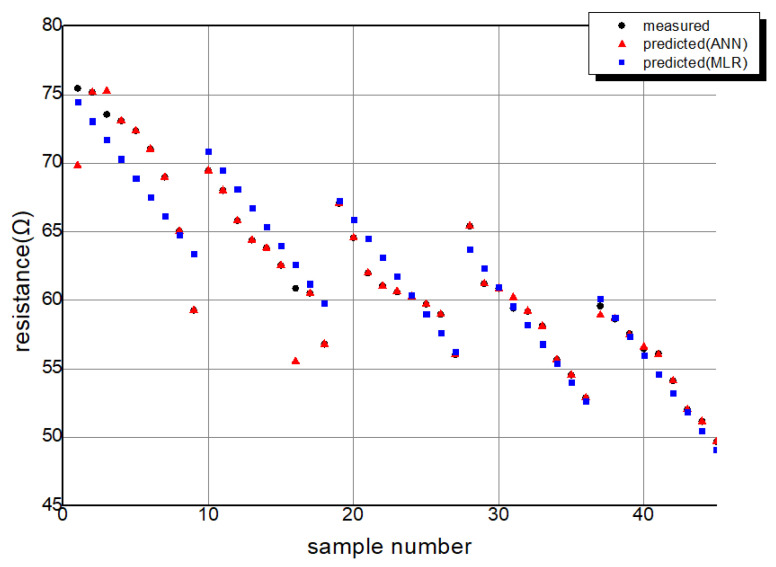
The distribution of the actual measured values and the values predicted by ANN, MLR.

The relationship between the predicted values and actual values for MLR and the ANN is depicted in Figure 11. In this analysis, 10% of the 45 input data points were randomly selected for testing. The MSE and coefficient of determination for the analysis using the ANN and MLR are provided in Table 3. Furthermore, the distribution of the actual measured values and the values predicted by the ANN and MLR for each sample can be seen in Figure 12, Figure 13 and Figure 14.

According to the experimental results, when predicting the resistance variation in conductive stitches using ANN, the coefficient of determination was 0.999 during training and 0.979 during testing, and the MSE reached a value of 0.00074. When using the MLR model, the coefficient of determination was 0.933, and the MSE value was 3.0503. This indicates a hit rate of 97.9% and 93.3% within the measured data, demonstrating high applicability for both models.

However, considering the coefficient of determination based on the testing data, the ANN outperformed MLR with a higher value of 0.979, which is 4.6 higher than MLR. This implies a more accurate prediction capability for the ANN.

Both models were able to predict the resistance based on the stitch length and angle of conductive stitches in wearable devices, thus reducing power loss. However, the optimized model using the ANN exhibited higher reliability. This demonstrates that using the trained ANN model can save time, costs, and materials while enabling the application of conductive stitches in various wearable fields.

Through experimental results, we observed that the width of the conductive thread increased as the stitch length decreased. Additionally, we observed that the resistance of the conductive thread varied with the stitch angle. Consequently, reducing the spacing between coated silver particles in conductive sewing led to decreased resistance and reduced power consumption. These results were closely related to the fabrication process of the conductive thread. The experimental silver-coated nylon yarn was coated using a silver paste solution. SEM observations revealed that the coated part of the conductive thread contained silver particles in a particulate state from the silver paste. This indicated a different perspective on resistance variation in conductive threads compared to conventional wires, as the electrical properties of conductive threads rely on the presence of silver particles on the surface acting as conductive agents. This also implies that the spacing between conductive particles (silver particles) can influence resistance, meaning that reducing the distance between conductive particles can lower resistance and subsequently reduce power consumption during power transfer due to conductivity. Moreover, as the stitch angle decreased and the stitch length increased, we observed that the tension applied to the thread increased, as evidenced by the measurement of the stitch width. As the tension increased, the gap between filaments and silver particles decreased, reducing power consumption.

These results hold significant importance in smart textiles, particularly from a wearable technology perspective. Conductive threads serve various roles in wearable technology applications, such as smart garments, health monitoring devices, and sports equipment. Conductive threads play a crucial role in power delivery and electrical signal transmission in wearable devices, especially in areas where power consumption should be minimized, such as in wearable energy harvesting. Additionally, high resistance in conductive threads may hinder signal transmission in electrical signal delivery applications, emphasizing the importance of reducing resistance to ensure proper functionality. In this study, we demonstrated that stitch patterns impact thread tension and, consequently, resistance, offering a means to reduce resistance in conductive threads through optimized stitch patterns. This finding has important implications for enhancing power delivery in wearable technology.

These results also extend beyond wearable technology into various other fields. Firstly, in the medical field, conductive threads find numerous applications in wearable medical devices and clothing for detecting and monitoring bio-signals, enabling real-time information for medical professionals to assess patient conditions. By understanding the impact of resistance changes in conductive threads, this research can improve the accuracy of detecting and interpreting bio-signals in medical applications. Secondly, in smart textiles, conductive threads offer possibilities in clothing, protective gear, and architectural materials. Smart clothing can perform various functions, such as temperature regulation, stress detection, and posture correction. Optimizing stitch patterns in conductive threads can enhance the performance of smart textiles and enable practical applications. Thirdly, in materials engineering, this research covers the process of creating conductive threads using silver-coated nylon fibers. Developing such processes and studying the interaction between silver particles and the thread can make valuable contributions to the field of materials engineering. Predicting and controlling resistance in conductive threads provides essential knowledge for practical applications in materials engineering, including predicting various fiber properties under different conditions, such as mechanical strength, durability, elasticity, and conductivity.

Furthermore, the integration of an ANN for prediction and its comparison with MLR holds significant implications. Notably, applying ANNs beyond the realm of wearable technology has several advantages. ANN models offer powerful tools to analyze and predict complex relationships within fiber systems, providing valuable research, development, and optimization insights. By utilizing ANNs, researchers can capture intricate patterns and nonlinear interactions in fiber characteristics, allowing for accurate prediction and optimization of various textile parameters. This includes predicting and optimizing fiber properties under various conditions, such as mechanical strength, durability, elasticity, and conductivity. Furthermore, ANN models aid in understanding the influence of various manufacturing processes on fiber properties, contributing to improved and controlled fiber quality and performance. The versatility of an ANN lies in its ability to learn from existing data and recognize underlying patterns to generalize predictions for unseen data. This makes it suitable for exploring new fiber science and technology areas, enabling the development of innovative materials, advanced textile structures, and intelligent fabrics. Integrating ANN-based prediction models in fiber manufacturing enhances process efficiency, improves product quality, and optimizes resource allocation. This enables a proactive decision-making process by predicting the expected performance of textile materials and guiding design choices for specific applications. Thus, it contributes to advancements in fashion, sportswear, technical textiles, and smart textiles.

In summary, utilizing ANNs for prediction in the field of fibers offers a powerful and versatile approach to improving understanding, control, and innovation within fiber systems. An ANN’s ability to capture complex relationships and make accurate predictions holds immense value for researchers, manufacturers, and designers in enhancing the textile industry. Predicting resistance changes in conductive threads allows for optimized stitch patterns, contributing to improved performance and extended battery life in wearable devices. Beyond wearable technology, this research can be applied in medical, smart textile, and materials engineering fields. Overall, ANN-based prediction in fibers and conductive threads has the potential to lead to the development of new technologies, products, and innovations, positively impacting related industries.

## 4. Conclusions

In this study, we observed the resistance changes in silver-coated conductive threads based on different stitch patterns and predicted these using MLR and ANN models. The tension applied to the conductive thread varied with stitch patterns, leading to changes in filament spacing. We observed that changes in spacing between coated silver particles influenced resistance. Longer stitch lengths resulted in reduced resistance, whereas larger stitch angles led to increased resistance. Based on these experimental findings, we optimized and compared the predictive results of both multiple linear regression (MLR) and artificial neural network (ANN) models. MLR analysis demonstrated that stitch length and angle were important variables affecting the resistance of the conductive thread. In comparison, the ANN model exhibited a superior resistance prediction performance over MLR analysis. The ability of the ANN to model complex interactions and nonlinearities allowed for more accurate predictions of resistance variations in conductive threads based on stitch patterns. Additionally, the ANN model demonstrated flexibility in optimizing the resistance prediction model through learning. Consequently, this study concluded that using an ANN for predicting the resistance of conductive threads based on stitch patterns was the most effective approach while acknowledging that the MLR model also provided efficient predictions. These results hold practical value in optimizing stitch patterns and controlling resistance in various applications that involve conductive threads.

Furthermore, this research provides valuable insights into the electrical and electronic industries and the textile and materials engineering fields. Understanding resistance variations in conductive threads and utilizing optimized models to enhance component performance can lead to innovation and advancements in multiple industries. Therefore, this study holds academic significance and demonstrates practical applicability in industrial sectors.

## Figures and Tables

**Table 1:** Variation in width according to the length of the sewing stitch.

Length of single sewing stitch	1 mm	2 mm	3 mm	4 mm	5 mm
Width of single sewing stitch	0.144 mm	0.135 mm	0.132 mm	0.128 mm	0.121 mm

**Table 2:** The resistance values measured for each stitch angle and stitch length.

Stitch Angle (°)	Stitch Length (mm)	Resistance (Ω)
180	1	75.46562
160	1	75.17814
140	1	73.57052
120	1	73.09852
100	1	72.37586
80	1	71.03588
60	1	68.98456
40	1	65.05209
20	1	59.27464
180	2	69.45544
160	2	68.00751
140	2	65.81475
120	2	64.39168
100	2	63.82584
80	2	62.55164
60	2	60.86941
40	2	60.51869
20	2	56.80353
180	3	67.06255
160	3	64.57084
140	3	61.98866
120	3	61.06079
100	3	60.60671
80	3	60.30651
60	3	59.71375
40	3	58.97164
20	3	56.02832
180	4	65.41049
160	4	61.21312
140	4	60.85553
120	4	59.41257
100	4	59.19793
80	4	58.13526
60	4	55.67235
40	4	54.55707
20	4	52.90243
180	5	59.58104
160	5	58.63749
140	5	57.56939
120	5	56.45359
100	5	56.1087
80	5	54.12524
60	5	52.04365
40	5	51.16842
20	5	49.68008

**Table 3:** MSE and R-squared values for the MLR and ANN models.

Model	Performance Criteria	
	MSE	$R^{2}$
MLR	3.0503	0.933
ANN	0.0007	0.979

## Data Availability

The data presented in this study are available on request from the corresponding author.
